# Reviewer acknowledgement 2014

**DOI:** 10.1186/s12931-015-0169-7

**Published:** 2015-02-03

**Authors:** Jan Lötvall, Reynold A Panettieri

**Affiliations:** Göteborg University, Vasagatan 33, Göteborg, 411 37 Sweden; University of Pennsylvania, 423 Guardian Dr #175, Philadelphia, PA 19104 USA

## Abstract

The editors of *Respiratory Research* would like to thank all of our reviewers who have contributed to the journal in Volume 15 (2014).

**Ritesh Agarwal**

India

**Anurag Agrawal**

India

**Alvar Agusti**

Spain

**Akira Akasawa**

Japan

**John Alcorn**

United States of America

**Stefano Aliberti**

Italy

**Catarina Almqvist**

Sweden

**Namasivayam Ambalavanan**

United States of America

**Yassine Amrani**

United Kingdom

**Tobias Ankermann**

Germany

**David Ann**

United States of America

**Sabina Antoniu**

Romania

**Hirokazu Arakawa**

Japan

**Elias Awji**

United States of America

**Mona Bafadhel**

United Kingdom

**Chunxue Bai**

China

**Joan A. Barbera**

Spain

**Esther Barreiro**

Spain

**Eric Bateman**

South Africa

**Frederic Batteux**

France

**Natalie Bauer**

United States of America

**Hasan Bayram**

Turkey

**Richard Beasley**

New Zealand

**Frederic Becq**

France

**Kai Michael Beeh**

Germany

**Christoph Beisswenger**

Germany

**Kylie Belchamber**

United Kingdom

**Salvador Bello**

Spain

**Saverio Bellusci**

Germany

**Claudia Benjamim**

Brazil

**Valerie Besnard**

France

**Tomoko Betsuyaku**

Japan

**Surya Bhatt**

United States of America

**Andrea Bianco**

Italy

**Kimberly Bigelow**

United States of America

**Charlotte Billington**

United Kingdom

**Mark Andrew Birrell**

United Kingdom

**Natalia Bogatcheva**

United States of America

**Charlotte Bolton**

United Kingdom

**Jessica Bon**

United States of America

**Marcel Bonay**

France

**James Bonner**

United States of America

**Maria Bonsignore**

Italy

**Serhat Bor**

Turkey

**Michael Borchers**

United States of America

**Lee Anthony Borthwick**

United Kingdom

**Ynuk Bosse**

Canada

**Joshua Boyce**

United States of America

**Ken Bracke**

Belgium

**Thomas Brand**

United Kingdom

**John Brehm**

United States of America

**James Bridges**

United States of America

**Volker Brinkmann**

Germany

**Mairi Brittan**

United Kingdom

**Pierre-Regis Burgel**

France

**Chris Burtin**

Belgium

**Raúl Cabrera-Rubio**

Spain

**Peter Calverley**

United Kingdom

**Gianna Camiciottoli**

Italy

**Giorgio Walter Canonica**

Italy

**Andre Cantin**

Canada

**Gaetano Caramori**

Italy

**Ciro Casanova**

Spain

**Peter Castaldi**

United States of America

**James Chalmers**

United Kingdom

**Ta-Chien Chan**

Taiwan

**Stephen Chan**

United States of America

**Divay Chandra**

United States of America

**Pascal Chanez**

France

**Nan-Shan Chang**

Taiwan

**Kenneth Chapman**

Canada

**Ping Chen**

China

**Alfredo Chetta**

Italy

**Wen Chin Chiang**

Singapore

**Nan Chiang**

United States of America

**Hideki Chiba**

Japan

**Marco Chilosi**

Italy

**Doo Hyun Chung**

South Korea

**Kian Fan Chung**

United Kingdom

**Juliana Cini Perry**

Brazil

**Antonio Clavenna**

Italy

**David Collie**

United Kingdom

**Marco Contoli**

Italy

**Alexandru Corlateanu**

Moldova

**Giuseppe Cornaglia**

Italy

**Massimo Corradi**

Italy

**Alessandro Corti**

Italy

**Julio Cortijo**

Spain

**Soraia Costa**

Brazil

**Harvey Coxson**

Canada

**Bruno Crestani**

France

**Gerard Curley**

Canada

**Ronald Dahl**

Denmark

**Jordi de Batlle**

France

**Javier de Miguel Díez**

Spain

**Monique Dehoux**

France

**Bart Dekkers**

Netherlands

**Stefano Del Giacco**

Italy

**Marion Delcroix**

Belgium

**Himanshu Desai**

United States of America

**Fabiano di Marco**

Italy

**Quoc Thai Dinh**

Germany

**Miguel Divo**

United States of America

**Ratko Djukanovic**

United Kingdom

**Cristian Dogaru**

Switzerland

**Louise Donnelly**

United Kingdom

**James Donohue**

United States of America

**David Douda**

United States of America

**Jo Douglass**

Australia

**Mark Dransfield**

United States of America

**Nickolai Dulin**

United States of America

**Daniel Dusser**

France

**Carsten Ehrhardt**

Ireland

**Marinos Elia**

United Kingdom

**Charles Emala**

United States of America

**Pierre Ernst**

Canada

**Santiago Ewig**

Germany

**Leonardo Fabbri**

Italy

**Leo Fabbri**

Italy

**Aurelie Fabre**

Ireland

**Jeremy Falk**

United States of America

**Rosa Faner**

Spain

**Harrison Farber**

United States of America

**Laszlo Farkas**

United States of America

**Bernie Fischer**

United States of America

**Rachel Foong**

Australia

**Evangelia Fouka**

Greece

**Frits Franssen**

Netherlands

**Elaine Fuertes**

Germany

**Trentham Furness**

Australia

**Judith Garcia-Aymerich**

Spain

**Marian Garcia-Nuñez**

Spain

**Amiq Gazdhar**

Switzerland

**Moyar Qing Ge**

United States of America

**Arthur Gelb**

United States of America

**Patrick Geraghty**

United States of America

**Peter Gibson**

Australia

**Stephan Glasser**

United States of America

**Rainer Gloeckl**

Germany

**John Godleski**

United States of America

**Felicia Goh**

Australia

**Neil Goldenberg**

Canada

**Nira Goldstein**

United States of America

**Marcin Golec**

Poland

**Ludwig Gortner**

Germany

**Reinoud Gosens**

Netherlands

**Harry Gosker**

Netherlands

**Christopher Goss**

United States of America

**Abdelilah Gounni**

Canada

**Lidwien Graat-Verboom**

Netherlands

**Catherine Greene**

Ireland

**Christoph Griessinger**

Germany

**Jochen Grommes**

Germany

**Johan Grooten**

Belgium

**Christophe Guignabert**

France

**Erich Gulbins**

Germany

**Sudhiranjan Gupta**

United States of America

**Robert Guzy**

United States of America

**Angela Haczku**

United States of America

**James Hagood**

United States of America

**Kathleen Haley**

United States of America

**David Hall**

United States of America

**Ian Hall**

United Kingdom

**Alf Hamann**

Germany

**Meilan Han**

United States of America

**Phil Hansbro**

Australia

**Corrine Hanson**

United States of America

**Louise Hecker**

United States of America

**Matt Hegewald**

United States of America

**Irene Heijink**

Netherlands

**My Helms**

United States of America

**Marc Hershenson**

United States of America

**Pieter Hiemstra**

Netherlands

**Yuji Higashimoto**

Japan

**Blanca Himes**

United States of America

**Toyohiro Hirai**

Japan

**Anne Holland**

Australia

**Raewyn Hopkins**

New Zealand

**Vegard Hovland**

Norway

**Reuben Howden**

United States of America

**Biao Hu**

United States of America

**Francois Huaux**

Belgium

**Alice Huertas**

France

**Wouter Huvenne**

Belgium

**Machteld Hylkema**

Netherlands

**Tomomi Ichikawa**

Japan

**Masakazu Ichinose**

Japan

**Stephane Illiano**

France

**Hiromasa Inoue**

Japan

**Satoru Ito**

Japan

**Kathryn Ivey**

United States of America

**Bogdan Jakiela**

Poland

**Tania Janaudis-Ferreira**

Canada

**Sam Janes**

United Kingdom

**An Soo Jang**

South Korea

**Matthew Jankowich**

United States of America

**Anan Jarab**

Jordan

**Gisli Jenkins**

United Kingdom

**Prescilla Vera Jeurink**

Netherlands

**Samithamby Jeyaseelan**

United States of America

**Kylie Johnston**

Australia

**Paul Jones**

United Kingdom

**Pavol Joppa**

Slovakia

**Joseph Jude**

United States of America

**Wolfgang Jungraithmayr**

Switzerland

**Eleanor Kable**

Australia

**Ralf Kaiser**

Germany

**Suresh Kalathil**

United States of America

**Ravi Kalhan**

United States of America

**Noor Kalsheker**

United Kingdom

**Richard Kanner**

United States of America

**Khalil Karimi**

Canada

**Harry Karmouty-Quintana**

United States of America

**Maya Karta**

United States of America

**Hideki Katsura**

Japan

**Prabha Kc**

United States of America

**Achsah Keegan**

United States of America

**Alexandra Kathrin Kiemer**

Germany

**Victor Kim**

United States of America

**Paul Kingma**

United States of America

**Gregory Kinney**

United States of America

**Uday Kishore**

United Kingdom

**Alex KleinJan**

Netherlands

**Daren Knoell**

United States of America

**Akira Koarai**

Japan

**Takashi Kohno**

Japan

**Jay Kolls**

United States of America

**Stephanie Korn**

Germany

**Laura Koth**

United States of America

**Angela Koutsokera**

Switzerland

**Matthew Kowgier**

Canada

**Susanne Krauss-Etschmann**

Germany

**James Kreindler**

United States of America

**Ramaswamy Krishnan**

United States of America

**Stefan Krüger**

Germany

**Vera Krymskaya**

United States of America

**Kuldeep Kumawat**

Netherlands

**Maciej Kupczyk**

Poland

**Ichiro Kuwahira**

Japan

**Matthew Lammi**

United States of America

**Larry Lands**

Canada

**Irene Lang**

Austria

**Gee W Lau**

United States of America

**Federico Lavorini**

Italy

**Aili Lazaar**

United States of America

**Jae Cheol Lee**

South Korea

**Petra Leidinger**

Germany

**Andrew Leitch**

United Kingdom

**Arne Leitte**

Germany

**Steve Leu**

Taiwan

**Julia Ley-Zaporozhan**

Germany

**Ning Li**

United States of America

**Christian Lino Cardenas**

United States of America

**Huiping Liu**

United States of America

**Jie Liu**

United States of America

**Lucille London**

United States of America

**Jose Luis Lopez-Campos**

Spain

**Matthew Loza**

United States of America

**Fabrizio Luppi**

Italy

**Barbara Lutey**

United States of America

**Kelvin MacDonald**

United States of America

**Roberto Machado**

United States of America

**Tania Maes**

Belgium

**Piero Maestrelli**

Italy

**Antoine Magnan**

France

**Sarah Maher**

United Kingdom

**Arnaud Mailleux**

France

**Susan Majka**

United States of America

**Patrick Mallia**

United Kingdom

**G. B. John Mancini**

Canada

**Peter Mancuso**

United States of America

**Nilam Mangalmurti**

United States of America

**Marco Mantero**

Italy

**Alessandro Marcon**

Italy

**Bernard Mari**

France

**Thomas Mariani**

United States of America

**Stacey Martiniano**

United States of America

**Kevin Mason**

United States of America

**Maria Gabriella Matera**

Italy

**Clinton Mathias**

United States of America

**Akio Matsuda**

Japan

**Shin Matsuoka**

Japan

**Gustavo Matute-Bello**

United States of America

**Robin McAnulty**

United Kingdom

**Sharon McGrath-Morrow**

United States of America

**Ailsa McKay**

United Kingdom

**Tricia McKeever**

United Kingdom

**Pamela McShane**

United States of America

**Damian Medici**

United States of America

**Davendra Mehta**

United States of America

**Asuncion Mejias**

United States of America

**Andrea Melani**

Italy

**Javier Milara**

Spain

**Henry Milgrom**

United States of America

**Ann Millar**

United Kingdom

**Laura Millares**

Spain

**Bruce Miller**

United States of America

**Lisa Miller**

United States of America

**Massimo Miniati**

Italy

**Stefan Minocchieri**

Switzerland

**Marc Miravitlles**

Spain

**Makoto Miyara**

France

**Hiroyuki Mochizuki**

Japan

**Seyed Javad Moghaddam**

United States of America

**Firdaus AA Mohamed Hoesein**

Netherlands

**Winfried Möller**

Germany

**Eduard Monso**

Spain

**Paolo Montuschi**

Italy

**Konosuke Morimoto**

Japan

**Rory Morty**

Germany

**Takashi Motegi**

Japan

**Kamal Mubarak**

United States of America

**Surafel Mulugeta**

United States of America

**Shigeo Muro**

Japan

**Lynne Murray**

United Kingdom

**Jean-Marc Naccache**

France

**Robert Naeije**

Belgium

**Per Nafstad**

Norway

**Meera Nair**

United States of America

**Luis Nannini**

Argentina

**Kristine Ng**

United Kingdom

**Richard Nho**

United States of America

**Linda Nici**

United States of America

**Heber Nielsen**

United States of America

**Jake Nikota**

Canada

**Rebecca Oberley-Deegan**

United States of America

**Paul O'Byrne**

Canada

**Toru Oga**

Japan

**Brian Oliver**

Australia

**Henric Olsson**

Sweden

**Hisamitsu Omori**

Japan

**Christian Opitz**

Germany

**Michael O'Reilly**

United States of America

**Victor Ortega**

United States of America

**Sebastian R. Ott**

Switzerland

**Jin O-Uchi**

United States of America

**Caroline Owen**

United States of America

**Pierluigi Paggiaro**

Italy

**Robert Paine**

United States of America

**Shi Pan**

United States of America

**Annie Pardo**

Mexico

**Kalpaj Parekh**

United States of America

**Samir Parikh**

United States of America

**Jin-Ah Park**

United States of America

**James Parker**

United States of America

**Martyn R. Partridge**

United Kingdom

**Bruce Patterson**

United States of America

**Markus Paulmichl**

Austria

**Ian Pavord**

United Kingdom

**Ray Stokes Peebles**

United States of America

**Gunnar Pejler**

Sweden

**Raymond Penn**

United States of America

**Tonio Pera**

United States of America

**Thierry Perez**

France

**Georgia Perona-Wright**

Canada

**Mark Perrella**

United States of America

**Marc Peters-Golden**

United States of America

**Charles Pilette**

Belgium

**Victor Pinto-Plata**

United States of America

**Laurent Plantier**

France

**Mathias Pletz**

Germany

**Francesca Polverino**

United States of America

**Maryam Poorafshar**

Sweden

**Martin Post**

Canada

**Dirkje Postma**

Netherlands

**Douglas Powell**

United States of America

**Matthew Poynter**

United States of America

**Alexa Pragman**

United States of America

**Y.S. Prakash**

United States of America

**Milo Puhan**

United States of America

**Marko Radic**

United States of America

**Madeleine Rådinger**

Sweden

**Julio Ramirez**

United States of America

**Shoshana Ravid**

Israel

**Ana Rebane**

Estonia

**Angelika Reissig**

Germany

**Elisabetta Renzoni**

United Kingdom

**Niki Reynaert**

Netherlands

**Paul Reynolds**

United States of America

**David Riches**

United States of America

**Nicolas Riteau**

United States of America

**Jesse Roberts**

United States of America

**Nicolas Roche**

France

**Jason Rock**

United States of America

**Roberto Rodriguez-Roisin**

Spain

**Micaela Romagnoli**

Italy

**Jesse Roman**

United States of America

**Freddy Romero**

United States of America

**Jan Rossaint**

Germany

**Nikoletta Rovina**

Greece

**Lewis Rubin**

United States of America

**Chen Rui**

China

**Clark Russell**

United Kingdom

**Erica Rutten**

Netherlands

**Jeong-Seon Ryu**

South Korea

**Ramsey Sabit**

United Kingdom

**Hironori Sagara**

Japan

**Umadevi Sajjan**

United States of America

**Cesare Saltini**

Italy

**Yan Sanders**

United States of America

**Thomas Sandstrom**

Sweden

**Federica Sangiuolo**

Italy

**Raul Sansores**

Mexico

**Elizabeth Sapey**

United Kingdom

**Kimio Satoh**

Japan

**Ian Sayers**

United Kingdom

**Francesco Scaglione**

Italy

**Mario Scartozzi**

Italy

**Dedmer Schaafsma**

Canada

**Andrea Schamberger**

Germany

**Tamara Schikowski**

Switzerland

**Sebastian Schmidt**

Germany

**Carsten Schmidt-Weber**

Germany

**Konrad Schultz**

Germany

**Jean-Clare Seagrave**

United States of America

**Terence Seemungal**

Trinidad and Tobago

**Susana Seixas**

Portugal

**Sanjay Sethi**

United States of America

**Maryam Sharifi-Sanjani**

United States of America

**Renat Shaykhiev**

United States of America

**Ganesh Shelke**

Sweden

**Rebecca Shilling**

United States of America

**Hiroaki Shimokawa**

Japan

**Janis Shute**

United Kingdom

**Nikos Siafakas**

Greece

**Patricia Silva**

Brazil

**Cherie Singer**

United States of America

**Dave Singh**

United Kingdom

**Sally Singh**

United Kingdom

**Sunit Singla**

United States of America

**Tea Skaaby**

Denmark

**Lidwien A. M. Smit**

Netherlands

**Sukhwinder Singh Sohal**

Australia

**Nestor Soler**

Spain

**Ronald Sorkness**

United States of America

**Giovanni Sotgiu**

Italy

**Paolo Spagnolo**

Italy

**Antonio Spanevello**

Italy

**Markus Sperandio**

Germany

**Peter Spieth**

Germany

**Domenico Spina**

United Kingdom

**Saranya Sridhar**

United Kingdom

**Michael Stenger**

United States of America

**Rob Stockley**

United Kingdom

**Daiana Stolz**

Switzerland

**Charlie Strange**

United States of America

**Eui-Sik Suh**

United Kingdom

**Samy Suissa**

Canada

**Maria Sukkar**

Australia

**Wayne Sullender**

United States of America

**Ying Sun**

United Kingdom

**Benjamin Suratt**

United States of America

**Nicola Sverzellati**

Italy

**Aleksandra Szczepankiewicz**

Poland

**Christoph Tabeling**

Germany

**Jun-Ming Tang**

China

**Laima Taraseviciene-Stewart**

United States of America

**Sadatomo Tasaka**

Japan

**Donald P Tashkin**

United States of America

**Christian Taube**

Netherlands

**Thomas Taylor-Clark**

United States of America

**Luc Teppema**

Netherlands

**Stefanos Theoharis**

Germany

**Mike Thomas**

United Kingdom

**Omar Tliba**

United States of America

**Yuji Tohda**

Japan

**Sara Tomassetti**

Italy

**Jean-Nicolas Tournier**

France

**Renata Trimer**

Brazil

**Maria Tsoumakidou**

Greece

**Argyris Tzouvelekis**

Greece

**Paola Ulivi**

Italy

**Lena Uller**

Sweden

**Omar Usmani**

United Kingdom

**John Ussher**

Canada

**Joost G. van den Aardweg**

Netherlands

**Thys van der Molen**

Netherlands

**Mart van der Plas**

Netherlands

**Lowie van Fleteren**

Netherlands

**Lindsey van Haute**

United Kingdom

**Carlo Vancheri**

Italy

**Jeroen Vanoirbeek**

Belgium

**Walter Verbrugghe**

Belgium

**Stijn Verleden**

Belgium

**Elcio Vianna**

Brazil

**Jordi Vilaro**

Spain

**Julio Villena**

Argentina

**Ross Vlahos**

Australia

**Claus Vogelmeier**

Germany

**Gerry Wagenaar**

Netherlands

**Alan Walker**

United Kingdom

**David Warburton**

United States of America

**Matthew Wargo**

United States of America

**Peter Wark**

Australia

**George Washko**

United States of America

**Jenora Waterman**

United States of America

**Christopher Waters**

United States of America

**James Michael Wells**

United States of America

**Tobias Welte**

Germany

**Fu-Qiang Wen**

China

**Geertjan Wesseling**

Netherlands

**Chris Weston**

United Kingdom

**Guillaume Wettstein**

France

**Jim White**

United States of America

**Martin Witzenrath**

Germany

**Michael Wolin**

United States of America

**Lisa Wood**

Australia

**Ashley Woodcock**

United Kingdom

**Reen Wu**

United States of America

**Ximei Wu**

China

**Richard Wunderink**

United States of America

**Wim Wuyts**

Belgium

**Ian Yang**

Australia

**Yunlei Yang**

United States of America

**Ozlem Yilmaz**

Turkey

**Peng Yin**

China

**Lan Yin**

China

**Elodie Zana-Taïeb**

France

**Emanuela Zannin**

Italy

**Bojan Zaric**

Serbia

**Joseph Zasadzinski**

United States of America

**Amir Zeki**

United States of America

**Lubo Zhang**

United States of America

**Linjie Zhang**

Brazil

**Weian Zhao**

United States of America

**Youyang Zhao**

United States of America

